# Multi-omics profiling of fungal balls in chronic pulmonary aspergillosis patients reveals microbiome dynamics and metabolic adaptations

**DOI:** 10.1128/mbio.00348-26

**Published:** 2026-05-05

**Authors:** Chan Liu, Matheus Mertz Ribeiro, Jian Yang, Liangyu Li, Jianxiong Li, Xianqiu Chen, Yude Wang, Le-Le Wang, Beibei Wang, Yiming Zhou, Jing Zhang, Jijin Jiang, Jielu Lin, Endrews Delbaje, Jin-Fu Xu, Gustavo H. Goldman, Shuo Liang

**Affiliations:** 1 Department of Respiratory and Critical Care Medicine, Shanghai Pulmonary Hospital, School of Medicine, Tongji University 12476, Shanghai, China; 2 Institute of Respiratory Medicine, School of Medicine, Tongji University, Shanghai, China; 3 Faculdade de Ciências Farmacêuticas de Ribeirão Preto, Universidade de São Paulo 28133, São Paulo, Brazil; 4 Department of Thoracic Surgery, Shanghai Pulmonary Hospital, School of Medicine,Tongji University 12476, Shanghai, China; 5 Department of Microbiology, Immunology, and Transplantation, KU Leuven 26657, Leuven, Belgium; 6 Department of Pathology, Shanghai Pulmonary Hospital, School of Medicine, Tongji University 12476, Shanghai, China; 7 Departamento de Biologia, Faculdade de Filosofia, Ciências e Letras de Ribeirão Preto, Universidade de São Paulo 28133, São Paulo, Brazil; 8 Department of Respiratory and Critical Care Medicine, Tongji Hospital, School of Medicine, Tongji University 12476, Shanghai, China; 9 National Institute of Science and Technology in Human Pathogenic Fungi, Ribeirão Preto, Brazil; Instituto Carlos Chagas, Curitiba, Brazil

**Keywords:** aspergilloma, chronic pulmonary aspergillosis, *Aspergillus fumigatus*, *Pseudomonas aeruginosa*, *Haemophilus influenzae*

## Abstract

Fungal balls (aspergillomas) are a debilitating complication of chronic pulmonary aspergillosis, but their functional biology as multi-kingdom ecosystems is poorly understood. Through integrated multi-omics analysis of 61 patient-derived fungal balls, we reveal their complex ecology. While *Aspergillus fumigatus* dominates the fungal niche (59% of patients), bacterial co-colonization is ubiquitous, primarily by *Pseudomonas aeruginosa* and *Haemophilus influenzae*. Metabolomics and metatranscriptomics unveil a structured division of labor and active warfare, including metabolic cross-feeding, competition for iron, and reciprocal antagonism via secondary metabolites, such as fumagillin and fumigaclavine C produced by *A. fumigatus*. Host metabolomics and transcriptomics revealed a potent but dysregulated human immune response, characterized by neutrophil activation and failed resolution. Our findings redefine aspergilloma not as a mere fungal aggregate, but as a resilient polymicrobial biofilm across kingdoms, in which synergistic and antagonistic inter-kingdom interactions drive pathogenesis and chronicity, suggesting new therapeutic strategies targeting the pathogenic consortium.

**IMPORTANCE:**

Chronic pulmonary aspergillosis (CPA) and its hallmark fungal balls (aspergillomas) represent a debilitating and difficult-to-treat respiratory disease, affecting millions worldwide. Here, we provide the first integrated multi-omics profile of surgically resected fungal balls from 61 CPA patients, revealing these structures not as mere fungal colonies, but as resilient, cross-kingdom biofilms teeming with bacterial co-colonizers, particularly *Pseudomonas aeruginosa* and *Haemophilus influenzae*. Our findings uncover a dynamic battlefield where fungi and bacteria engage in metabolic cross-feeding, chemical warfare, and competition for nutrients such as iron. We demonstrate that the host mounts a potent but dysregulated immune response characterized by chronic neutrophilic inflammation and failed resolution, driving tissue damage and disease persistence. Our data provide a foundation for novel therapeutic strategies aimed at disrupting microbial synergy, modulating host inflammation, and breaking the cycle of chronic infection, an approach that could significantly improve outcomes for patients with this refractory disease.

## INTRODUCTION

Chronic pulmonary aspergillosis (CPA) is a devastating, progressive infectious syndrome caused by ubiquitous *Aspergillus* species, affecting an estimated 1.8 million people annually worldwide (1, 2). The disease characteristically arises in individuals with pre-existing structural lung disease, such as cavities left by tuberculosis or chronic obstructive pulmonary disease (COPD) (3). Unlike invasive aspergillosis, which predominantly affects severely immunocompromised individuals, CPA occurs in patients who are largely immunocompetent or in those with mild immunocompromise (4, 5). A hallmark of CPA is the formation of fungal balls (aspergillomas) within lung cavities. Critically, these are not mere aggregates of hyphae but complex, structured biofilms: well-circumscribed masses consisting of a dense extracellular matrix that entangles fungal hyphae, inflammatory cells, and host-derived debris (6). This biofilm mode of growth is not benign; it is a nexus of pathology, driving persistent inflammation, progressive lung destruction, and life-threatening hemoptysis (7). The biofilm matrix confers inherent therapeutic recalcitrance, creating a sanctuary for the evolution of antifungal resistance and contributing to recurrent relapses, which collectively complicate long-term disease management (8–10).

Central to CPA pathogenesis is the pulmonary cavity, which functions not merely as a passive space but as a dynamic microenvironment fostering fungal-bacterial interactions and biofilm maturation. Increasing clinical evidence suggests a complex relationship between fungi and bacteria within these cavities. The lung cavities themselves provide an ideal niche for bacterial colonization by pathogens such as *Pseudomonas aeruginosa*, *Haemophilus influenzae*, and *Staphylococcus aureus* (11). Simultaneously, severe bacterial infections, including necrotizing pneumonia, can lead to cavity formation, creating an environment conducive to fungal invasion and subsequent biofilm development into a mature aspergilloma (7, 12). This bidirectional relationship highlights the frequent coexistence of fungi and bacteria. It emphasizes the importance of considering their interactions—potentially within a shared biofilm architecture—rather than treating them as separate events. Aspergillomas therefore develop as biofilms within a polymicrobial context, where the specific microbial ecology, inter-kingdom interactions, and metabolic adaptations driving fungal biofilm persistence remain largely unexplored.

Recent advances in microbiome science have transformed our understanding of the lung from a sterile organ to a dynamic microbial ecosystem, where fungi coexist with diverse bacterial communities and interact with host immunity (13, 14). Studies in cystic fibrosis (CF) and chronic obstructive pulmonary disease (COPD) have demonstrated that inter-kingdom interactions—particularly between *Aspergillus fumigatus*, *Candida* species, and opportunistic bacteria like *P. aeruginosa*—play a critical role in disease progression and biofilm formation (15). These interactions can be antagonistic, synergistic, or metabolic in nature, influencing fungal growth, biofilm architecture, and host immune responses (16–18). Notably, metabolic exchanges involving amino acids, iron, and tryptophan derivatives have been implicated in microbial adaptation, biofilm matrix production, and immune modulation within the lung niche (19, 20). However, the extent to which these microbiome dynamics and metabolic adaptations define the biofilm ecology of the fungal ball in CPA remains poorly investigated.

Compounding this complexity is the influence of fungal secondary metabolism and host-microbiota co-metabolism on biofilm resilience. Genome-scale metabolic models of *A. fumigatus* have identified strain-specific dependencies on aromatic amino acid pathways and host- or microbiota-derived metabolites, which may serve as building blocks for biofilm matrix components (21). Additionally, metabolomic profiling in respiratory diseases has revealed altered tryptophan and phenylalanine catabolism, which are linked to interleukin-22-mediated mucosal immunity and immune tolerance (22, 23). Together, these findings underscore the need for integrated approaches that move beyond single-pathogen perspectives to capture the multi-layered ecological and metabolic interactions that drive fungal biofilm persistence *in vivo*.

Dissecting such a complex, spatially organized biofilm microenvironment is intractable with conventional, culture-based methods, which fail to capture the full breadth of microbial diversity and *in situ* activity. A multi-omics approach is therefore essential to deconstruct and characterize the aspergilloma biofilm ecosystem. By integrating 16S rRNA and ITS amplicon sequencing (community structure), metatranscriptomics (active gene expression), and metabolomics (the chemical phenotype), we can obtain a systems-level understanding of this pathogenic niche. Such an integrated strategy, although transformative in other fields of microbiology, has never been applied to the CPA fungal ball. Previously, there were two reports on the metabolomics of fungal ball sinusitis and paranasal sinuses in several patients, but no information was provided about the species identification of the organisms colonizing the fungal ball (24, 25). However, fungal and bacterial diversity of sinus samples from 38 patients with Fungal Ball Rhinosinusitis (FBRS) showed that *A. fumigatus* and *H. influenzae* were the main fungal and bacterial genera identified in FBRS (26). Here, we present the first multi-omic characterization of aspergillomas resected from CPA patients. We sought to delineate the taxonomic and functional architecture of the fungal ball biofilm microbiome and uncover the active transcriptional networks of *A. fumigatus*, its microbial partners, and human cells, identifying the metabolic exchanges that underpin their coexistence and persistence. This work redefines the aspergilloma as a complex polymicrobial biofilm ecosystem**,** reveals key metabolic adaptations and vulnerabilities of clinical *A. fumigatus* strains within their native environment, and provides a new conceptual framework for developing therapies that target the pathogenic consortium and its resistant biofilm state rather than the fungus in isolation.

## RESULTS

### Characterization of the cohort of 61 Chinese patients

**Fig 1:**
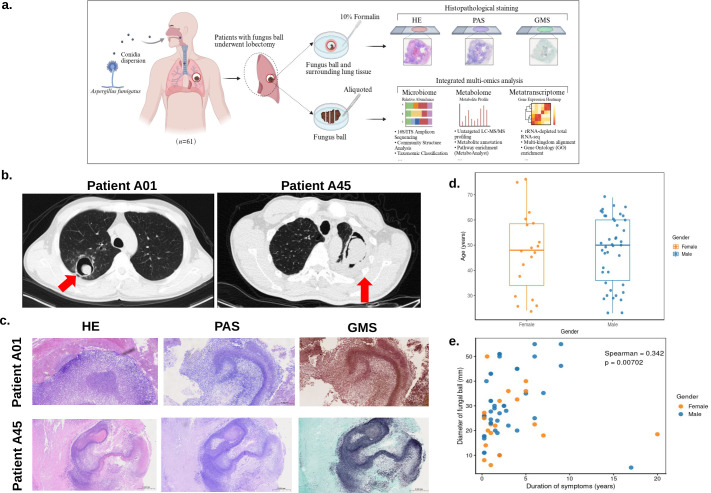
Multi-omics analysis of a cohort of 61 fungal balls surgically operated on in the lobectomy procedure. Experimental overview, demographic distribution, and clinical correlation of aspergilloma samples. (**a**) Schematic representation of the experimental design. Clinical material from patients diagnosed with pulmonary aspergilloma was surgically collected, divided into independent aliquots, and subjected to multiomics analyses: microbiome (16S/ITS amplicon-seq), metatranscriptome (mNGS), untargeted metabolomics (LC-MS/MS), and histopathology. The data obtained at each omics layer were subsequently integrated to characterize the molecular and metabolic mechanisms associated with *A. fumigatus* infection. Biorender was used to draw the figure. (**b**) Computed tomography (CT) scan to visualize fungal masses and disease within the lungs of patients A45 and A50. (**c**) Lung histopathology of patients A45 and A50. HE, hematoxylin and eosin; PAS, periodic acid-Schiff; GMS, Grocott methenamine silver. Bars: 0.5 mm. (**d**) Age distribution of patients included in the study, stratified by sex (males in blue and females in orange). The dispersion and median values are presented as boxplots. (**e**) Correlation between fungus ball diameter and symptom duration, broken down by sex. A positive, statistically significant correlation was observed between the two variables (Spearman’s ρ = 0.34, *P* = 0.007), suggesting a progressive increase in lesion diameter with increasing symptomatic chronicity and a possible distinct clinical pattern between men and women.

Sixty-one patients clinically diagnosed with chronic pulmonary aspergillosis (CPA) were enrolled, surgically operated on (lobectomy procedure), and discharged between January 2020 and December 2024 from the Shanghai Pulmonary Hospital, Shanghai, China (see Tables S1 and S2 at https://doi.org/10.6084/m9.figshare.31281223). The cohort included 42 males and 19 females, with a wide age range (Fig. 1d; Table S1 at https://doi.org/10.6084/m9.figshare.31281223). Hemoptysis was the most common presenting symptom (72%). Key underlying conditions included bronchiectasis (51%), a history of tuberculosis (21%), chronic obstructive pulmonary disease (COPD, 10%), allergic bronchopulmonary aspergillosis (ABPA, three patients), and asthma (two patients) (Table S1 at https://doi.org/10.6084/m9.figshare.31281223).

Once admitted, most patients underwent pre-operative sputum culture, the (1,3)-β-D-glucan test (G test), and the galactomannan (GM) antigen test (see Table S2 at https://doi.org/10.6084/m9.figshare.31281223). Among the 61 patients, 38 (62%) showed positive microbiological or serological evidence of *Aspergillus* infection prior to surgery (Table S2, https://doi.org/10.6084/m9.figshare.31281223). Notably, postoperative histopathology confirmed the presence of filamentous fungi in all 61 resected specimens, validating their inclusion in the analysis. Computed tomography (CT) scans and histopathology were used to visualize fungal masses and infection in the lungs of selected patients (Fig. 1b and c; Fig. S1 and S2, https://doi.org/10.6084/m9.figshare.31281223). Although a negative GM test result does not entirely rule out CPA or an *Aspergillus* infection, these pre-operative results suggest the possible fungal infection by non-*Aspergillus* species. This highlights the need for precise molecular identification to characterize the true composition of these clinically diagnosed fungal balls.

To investigate the fungal ball microbiome, metabolome, and metatranscriptomics, the excised fungal balls (54% from the left lobe and 46% from the right lobe) were collected, partly sent for pathological staining, and partly preserved for multi-omics analysis (Fig. 1a; see Table S1 at https://doi.org/10.6084/m9.figshare.31281223). A total of 33 clinical and laboratory features were evaluated (Table S1 and Fig. S3a, https://doi.org/10.6084/m9.figshare.31281223). A weak but statistically significant correlation was detected between the diameter of the fungal ball (mm) and the duration of symptoms (Spearman’s ρ = 0.34, *P* = 0.007; Fig. 1e; Table S1, https://doi.org/10.6084/m9.figshare.31281223). No other significant correlations were detected among these variables.

### Identification of the fungal and bacterial populations in the fungal balls

**Fig 2:**
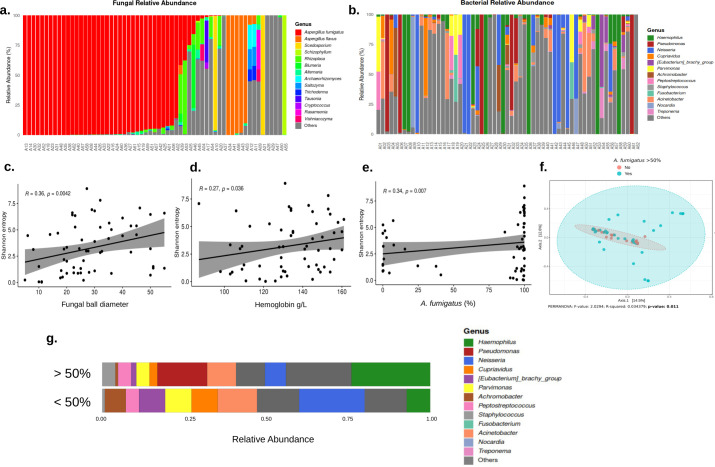
Identification of bacteria and fungi that colonize the 61 fungal balls. Relationships between fungal and bacterial diversity and clinical parameters in patients with pulmonary aspergilloma. (**a**) Relative abundance of the main fungal species detected by amplicon sequencing (ITS1), represented on the *y*-axis and distributed by sample on the *x*-axis. Each bar corresponds to an individual sample and illustrates the fungal taxonomic composition among patients. (**b**) Relative abundance of bacterial species (16S rRNA) in the same samples, demonstrating the variation in the bacterial microbiota associated with the disease. (**c**) Relationship between fungal ball diameter and microbial diversity (Shannon entropy). Each point represents a patient, with the coefficient of determination (*R*²) and *P*-value indicated on the graph, highlighting the correlation between lesion size and microbial community complexity. (**d**) Correlation between hemoglobin levels (g L⁻¹) and Shannon diversity. The linear fit and statistical values (*R*² and *P*) are shown, indicating a possible association between hematologic status and lung microbial diversity. (**e**) Association between the relative proportion of *A. fumigatus* and Shannon’s entropy. Fungal dominance by *A. fumigatus* is associated with increased local community diversity. (**f**) Principal component analysis (PCA) of microbial communities, distinguishing patients with high (>50%) and low (<50%) relative abundance of *A. fumigatus*. The spatial separation of the groups indicates divergence in the microbial structure associated with fungal dominance. (**g**) Relative abundance of the main bacterial species comparing patients with infections dominated by *A. fumigatus* (>50%) and those with lower predominance (<50%). The analysis seeks to identify patterns of co-occurrence and potential synergistic or antagonistic interactions between *A. fumigatus* and concomitant bacterial species.

The composition of the microbiomes was analyzed using 16S rDNA and ITS sequencing. Analysis of beta diversity, expressed as the relative abundance of bacterial species, revealed highly variable fungal and bacterial composition across patients (Fig. 2a and b). Among the 61 fungal balls, 49 (80.3%) were dominated by a single fungal species (>90% composition), while the remaining 12 contained mixed species. The dominant species in these 49 samples were *A. fumigatus* (36 cases, 59% of all patients), *A. flavus* (5 patients), *Scedosporium* spp. (2 patients), and unidentified species (6 patients) (Fig. 2a). The bacteria that comprise more than 90% of the bacterial species are as follows: 6 of *Haemophilus influenzae*, 5 of *Pseudomonas aeruginosa*, 6 of *Neisseria*, 1 of *Nocardia*, 10 of unidentified species, and 33 of mixed species (Fig. 2b). It is important to notice that we defined unidentified species mainly based on the database limitations/resolution of the amplicon sequencing. For some bacterial and fungal species, identity was confirmed by phylogenetic analysis (see Fig. S4, S5 and Table S3 at https://doi.org/10.6084/m9.figshare.31281223). Bacterial diversity was positively correlated with the diameter of the fungal ball (*R* = 0.36, *P* = 0.0042), the relative abundance of *A. fumigatus* (*R* = 0.27, *P* = 0.036), and patients’ hemoglobin levels (*R* = 0.34, *P* = 0.007) (Fig. 2c through e). However, it is not clear if the correlation between bacterial diversity and fungal ball diameter reflects longer infection time, allowing for more complex colonization, or whether a more diverse community promotes growth. Although bacterial communities associated with *A. fumigatus* exhibited high inter-patient dissimilarity, *P. aeruginosa* showed an influential association with *A. fumigatus* (*R* = 0.34, *P* = 0.01) (Fig. 2f). We examined potential associations between the main bacterial taxa and five clinical conditions using Phi correlation coefficients (Fig. S3b, https://doi.org/10.6084/m9.figshare.31281223). The results showed consistently weak correlations, with an average absolute value of only 0.12 (Fig. S3b, https://doi.org/10.6084/m9.figshare.31281223). Of 40 comparisons, only one reached conventional significance, and it would not hold up after adjustment for multiple testing (Fig. S3b, https://doi.org/10.6084/m9.figshare.31281223). Overall, the data do not support a systematic link between the bacterial taxa assessed and the clinical conditions studied. Collectively, these findings indicate that fungal balls harbor bacterial and fungal diversity, with a marked predominance of *A. fumigatus, P. aeruginosa*, and *H. influenzae*.

### Metabolomic profiling of *A. fumigatus* fungal balls reveals a cross-kingdom metabolic network driving pathogenesis

**Fig 3:**
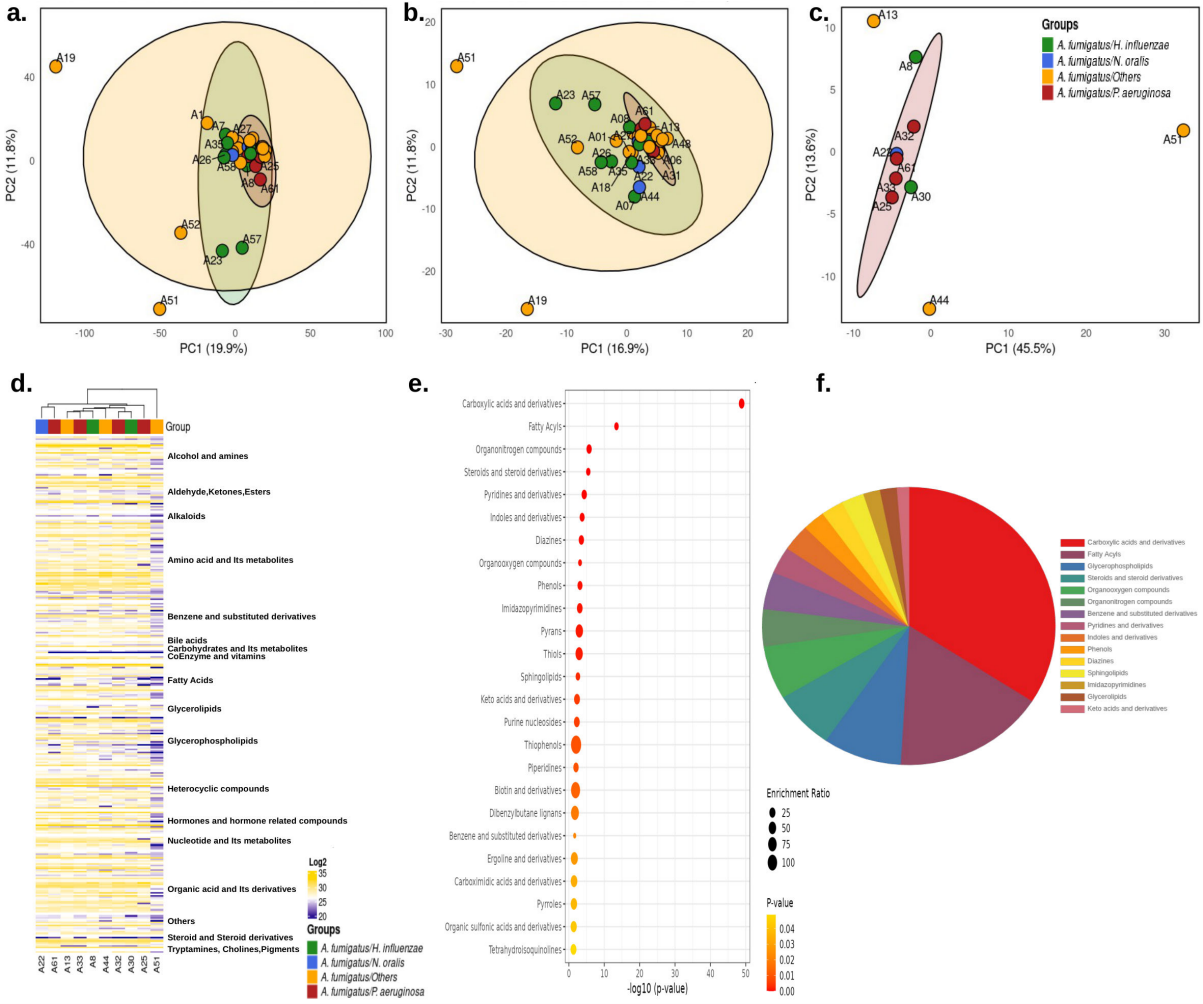
Integrative metabolomic analysis of microbial associations in pulmonary aspergilloma. Principal component analysis (PCA) of metabolomic profiles obtained by LC-MS/MS for (**a**) 34 patients with 3,417 metabolites, (**b**) 34 patients with 300 metabolites, and (**c**) 10 patients with 300 metabolites, demonstrating the distribution of metabolites produced by different microbial associations identified in the disease. Each point represents a sample, colored according to the dominant consortium: *A. fumigatus/H. influenzae* (green), *A. fumigatus/N. oralis* (blue), *A. fumigatus*/Others (yellow), and *A. fumigatus/P. aeruginosa* (red). The separation of the clusters suggests distinct metabolic signatures associated with different fungal-bacterial interactions. (**d**) Heatmap of the 300 most discriminating metabolites (converted to log₂ values), representing the variation in intensity between samples. The color scales range from blue (lowest relative abundance) to yellow (highest relative abundance). Metabolites are categorized according to their predominant origin: bacterial, fungal, human, or ubiquitous. (**e**) Pathway enrichment analysis of the top 25 metabolic pathways identified among the 300 selected metabolites (*P* < 0.05). (**f**) Distribution of the chemical classes of the 300 selected metabolites, grouped according to categories of lipids, organic acids, amino acids, nucleotides, and phenolic compounds. The analysis highlights the structural and functional diversity of metabolites involved in microbial interactions and host response.

Fungal balls are complex structures composed primarily of fungal hyphae, along with bacterial communities, host cells, and cellular debris. To investigate the biochemical interplay between these components, we performed untargeted metabolomic profiling via liquid chromatography-mass spectrometry (LC-MS) on 34 fungal ball samples. These samples were predominantly colonized by *A. fumigatus* (>90%; Fig. 2a; see Table S3 at https://doi.org/10.6084/m9.figshare.31281223). Our analysis identified a diverse array of metabolites, which we annotated using pathway enrichment, isotopic labeling, and spectral matching against reference databases (PubChem, HMDB, Metlin, and KEGG; Table S4, https://doi.org/10.6084/m9.figshare.31281223). We further classified the 300 most abundant compounds by their inferred producers and chemical classes (Fig. 3; Table S4, https://doi.org/10.6084/m9.figshare.31281223). The metabolites reflect contributions from human host metabolism and bacterial and fungal biosynthetic pathways, highlighting symbiotic, competitive, and host-pathogen interactions.

Principal component analysis based on 3,417 metabolites from these 34 samples revealed clear clustering trends among the different groups of *A. fumigatus* co-cultured with distinct bacterial species (Fig. 3a). The first two principal components explained 31.7% of the total variance (PC1 = 19.9%; PC2 = 11.8%), indicating that the metabolic variability is distributed across multiple dimensions. Samples corresponding to the *A. fumigatus/H. influenzae* co-culture formed a more compact cluster, suggesting a relatively homogeneous metabolic profile within this interaction. In contrast, *A. fumigatus/*Others showed greater dispersion and outliers (A19, A51, and A52), reflecting higher metabolic heterogeneity, possibly associated with the broader diversity of bacterial species in this group (Fig. 3a). Co-cultures of *Neisseria* sp. and *P. aeruginosa* exhibited intermediate metabolic profiles, partially overlapping with those of other groups, suggesting that while bacterial partners modulate fungal metabolism, there is no complete metabolic segregation among the consortia analyzed (Fig. 3a). When PCA was performed using 300 manually filtered metabolites, a similar clustering pattern was observed, with outliers primarily belonging to the Others group, a central cluster dominated by *A. fumigatus/H. influenzae*, and a partially overlapping group formed by *P. aeruginosa* and Others (Fig. 3b). However, this reduced data set also explained only a relatively small portion of the total variance (PC1 = 16.0%; PC2 = 11.8%), reinforcing the notion that metabolic differentiation among co-cultures is subtle and multidimensional.

To profile the metabolic landscape of bacterial co-colonization in *A. fumigatus* fungal balls, we selected 10 representative patients from a cohort of 61. The selection was designed to capture the spectrum of *A. fumigatus* (more than 95% prevalence) and bacterial colonization states, specifically (i) near-monoculture (>80% single species), (ii) mixed colonization with a dominant species (>40%), and (iii) polymicrobial colonization with no single dominant species. Thus, the following patients were chosen: (i) A8 and A30 (colonized by more than 95% *H. influenzae*), (ii) A25, A32, and A61 (colonized by more than 80% *P. aeruginosa*), (iii) A33 (colonized by more than 40% *P. aeruginosa*, and different proportions of *Acinetobacter*, *Staphylococcus*, *Parvimonas*, *Neisseria*, and *Haemophilus*), (iv) patient A22 (colonized by more than 95% *N. oralis*), (v) A44 (colonized by *N. subflava*, *Haemophilus*, *Peptostreptococcus*, *Staphylococcus*, *Acinetobacter*, and unidentified species), (vi) A51 (colonized by more than 40% *Acinetobacter piitti*, and different proportions of *Haemophilus*, *Peptostreptococcus*, and *Parvimonas*), and (vii) A13 (colonized by several bacteria, including *Nocardia* sp, *Parvimonas* sp, *Staphylococcus* sp, *Haemophilus* sp, and unidentified bacterial species). Similar to the PCA analysis of the 34 patient fungal balls (Fig. 3a and b), PCA analysis showed a high correlation among patients with *A. fumigatus* fungal balls colonized by *H. influenzae*, *N. oralis*, and mainly *P. aeruginosa* (Fig. 3c). MetaboAnalyst (https://www.metaboanalyst.ca/) revealed a significant enrichment of metabolites belonging to the classes of carboxylic acids and derivatives, fatty acids, glycerophospholipids, and steroids and steroid derivatives (*P* < 0.05; Fig. 3d through f; see Table S4 at https://doi.org/10.6084/m9.figshare.31281223).

*A. fumigatus* deploys a versatile metabolic arsenal for nutrient acquisition, chemical warfare, and stress tolerance. Analysis of the fungal-specific metabolome indicated that *A. fumigatus* functions as a versatile saprotroph, actively remodeling its environment. We detected a signature of intense proteolysis and amino acid catabolism, including metabolites of the tryptophan degradation pathway (L-kynurenine, indole-3-lactic acid) and cyclic dipeptides (diketopiperazines), indicating the breakdown of host tissues (see Table S4 at https://doi.org/10.6084/m9.figshare.31281223; Fig. 3c). Concurrently, fatty acid ethyl esters (ethyl hexadecanoate) and TCA cycle intermediates (3-hexenedioic acid) suggested active lipid utilization. We identified secondary metabolites, such as the potent anti-angiogenic agent fumagillin and the ergot alkaloid fumigaclavine C, consistent with a strategy to modulate the host response and suppress bacterial competitors (Table S4, https://doi.org/10.6084/m9.figshare.31281223; Fig. 3c). The presence of compatible solutes (betaine and erythritol) indicated robust fungal adaptation to oxidative stress, hypoxia, and a commitment to biofilm growth within the hostile host environment.

We identified key metabolites consistent with *P. aeruginosa*’s role as a primary degrader of complex substrates. The central intermediate of the β-ketoadipate pathway, 3-oxoadipic acid, along with phenol, demonstrated active breakdown of aromatic compounds, likely derived from host tissue (see Table S4 at https://doi.org/10.6084/m9.figshare.31281223; Fig. 3c). Concurrently, the presence of D-ornithine pointed to active biofilm remodeling by *P. aeruginosa*. We detected N-acetyl-L-alanine, a product of peptide degradation, suggesting cross-feeding of simpler carbon sources. In return, the obligate human pathogen *H. influenzae* contributed vitamin precursors, evidenced by a high abundance of 5,6-dimethylbenzimidazole, the dedicated precursor for vitamin B₁₂ (Table S4, https://doi.org/10.6084/m9.figshare.31281223; Fig. 3c). The co-occurrence of biotin, which *H. influenzae* cannot synthesize, highlights a dependency on the community or host, underscoring a tightly integrated metabolic niche.

The metabolomic signature was consistent with a chronic, non-growing but metabolically active bacterial population. We detected D-amino acids (D-ornithine and D-proline), which are incorporated into the peptidoglycan of stationary phase cells to promote antibiotic tolerance (see Table S4 at https://doi.org/10.6084/m9.figshare.31281223; Fig. 3c). The fatty acid biosynthesis intermediate (2E)-decenoyl-ACP indicated active, but slow, membrane turnover, while glutathionylspermidine suggested ongoing mitigation of host-derived oxidative stress (Table S4, https://doi.org/10.6084/m9.figshare.31281223; Fig. 3c). The consortium was defined by active chemical warfare. We detected bacterial antifungal agents, including the polyenes natamycin and tetromycin C5, indicating a direct assault on the fungal hyphae (Table S4, https://doi.org/10.6084/m9.figshare.31281223; Fig. 3c). It is important to note that none of these bacterial agents or derivatives were prescribed to the patients (Table S1, https://doi.org/10.6084/m9.figshare.31281223). The presence of 1,4-dihydroxy-2-naphthoate, a precursor to menaquinone (vitamin K₂), suggests additional pathways for bacterial cofactor synthesis with potential immunomodulatory effects on the host (Table S4, https://doi.org/10.6084/m9.figshare.31281223; Fig. 3c).

The host metabolome reflects a state of chronic inflammatory siege and systemic metabolic dysregulation. The host metabolic signature was consistent with a sustained, destructive, and non-resolving immune response. We observed a profound upregulation of pro-inflammatory lipid mediators, including leukotriene C4 (LTC4) and prostaglandins (PGD2, PGE3, PGB2, and PGA2), indicating persistent activation of neutrophils and macrophages (see Table S4 at https://doi.org/10.6084/m9.figshare.31281223; Fig. 3c). Direct evidence of oxidative damage was provided by elevated levels of o-tyrosine (protein damage) and allantoin (urate oxidation). The accumulation of collagen breakdown products (4-hydroxyproline) and sphingolipid signaling molecules (sphinganine) pointed to ongoing tissue remodeling and cell death (Table S4, https://doi.org/10.6084/m9.figshare.31281223; Fig. 3c). The infection induced a significant systemic burden, evidenced by markers of hepatic stress (altered bile acids and succinylacetone) and renal dysfunction (elevated symmetric dimethylarginine [SDMA] and creatinine) (Table S4, https://doi.org/10.6084/m9.figshare.31281223; Fig. 3c). The presence of neuroactive compounds (dopamine and endomorphin-2) suggested widespread neuro-immune crosstalk.

We identified a core set of metabolites that constitute a shared biochemical interface between the host and the microbial consortium, indicating that the biofilm functions as an integrated metabolic unit. Metabolites of universal biomass breakdown were highly abundant. These included dihydrouracil (nucleic acid catabolism), N-acetylated amino acids (N-acetyl-L-phenylalanine and protein turnover), and L-methionine sulfoxide (oxidative protein damage) (see Table S4 at https://doi.org/10.6084/m9.figshare.31281223; Fig. 3c). This indicates a continuous cycle where host-derived macromolecules are degraded into a shared nutrient pool. Host-derived complex lipids, such as the triglyceride TG(15:0/22:6/20:5), appeared to serve as a primary nutrient reservoir. The widespread presence of free fatty acids (myristic acid), their methyl esters, and phospholipid precursors (phosphatidic acid) indicated active lipid processing by all organisms (Table S4, https://doi.org/10.6084/m9.figshare.31281223; Fig. 3c). Key precursors of universal biosynthetic pathways were identified, most notably isopentenyl pyrophosphate (IPP), the building block for sterols (human cholesterol and fungal ergosterol). The presence of N6-acetyl-L-lysine suggested potential disruption of host epigenetic regulation through interference with protein acetylation states.

Overall, these connections illustrate a tripartite ecosystem in which human metabolites reveal a highly inflammatory environment that fuels microbial growth. At the same time, bacteria provide fermentation products for energy, and fungi contribute defensive compounds.

### Scanning electron microscopy reveals the polymicrobial biofilm architecture of *A. fumigatus* fungal balls

**Fig 4:**
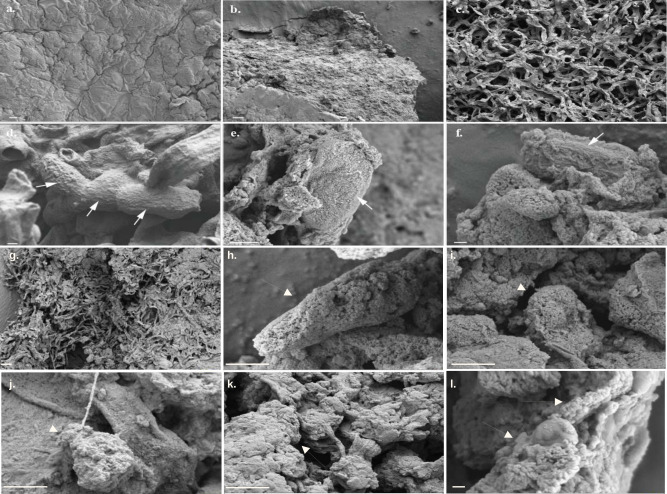
Scanning electron microscopy (SEM) reveals the polymicrobial biofilm architecture of pulmonary fungal balls. (**a to f**) Representative SEM images from a fungal ball specimen from patient A3 (colonized by *A. flavus*). (**a**) The outermost surface shows a dense, pellicle-like matrix. (**b**) A sub-surface region with fungal hyphae enmeshed in an extensive extracellular matrix (ECM) that incorporates host-derived components. (**c**) The core of the aspergilloma is composed of a dense, interconnected hyphal network forming the biofilm’s structural scaffold. (**d to f**) High-magnification views revealing bacterial cells (white arrowheads) in close physical association with fungal structures, either directly adhering to hyphal surfaces (**d and f**) or embedded within the surrounding ECM (**e**).(**g to l**) Representative SEM images from a fungal ball specimen from patient A33 (colonized by *A. fumigatus* and *P. aeruginosa*). (**g**) Low-magnification overview of the dense fungal core. (**h to l**) High-magnification views demonstrating intimate bacterial-fungal co-association, with bacterial morphotypes (white arrowheads) adhering to hyphae and embedded in the matrix, visually confirming a polymicrobial biofilm community. Scale bars: 2 µm (**a**), 10 µm (**b**, **c**, **g**), 1 µm (**d**, **e**,** h to k**), and 200 nm (**f and l**).

To elucidate the structural organization of the pulmonary fungal ball, we performed scanning electron microscopy (SEM) on surgically resected specimens from patients A3 and A33 (colonized by *A. flavus*, *A. fumigatus,* and *P. aeruginosa*, respectively) and selected patients A1 and 45 (see Fig. S6 at https://doi.org/10.6084/m9.figshare.31281223). Our analysis revealed a complex, multi-layered biofilm architecture that provides a physical context for the dynamic interkingdom interactions identified by our multi-omics approach. The outermost surface of the fungal ball was characterized by a dense, pellicle-like matrix that formed a protective barrier for the underlying community (Fig. 4a; Fig. S6, https://doi.org/10.6084/m9.figshare.31281223). Beneath this surface, we observed an intricate meshwork of fungal hyphae extensively enmeshed within a robust extracellular matrix (ECM) (Fig. 4b; Fig. S6, https://doi.org/10.6084/m9.figshare.31281223). This ECM aggregated host-derived components, including inflammatory cells and cellular debris, corroborating the metabolomic findings of intense host tissue breakdown. The core of the aspergilloma was composed of a dense, interconnected network of hyphae, forming a structural scaffold characteristic of a mature biofilm (Fig. 4c and g; Fig. S6, https://doi.org/10.6084/m9.figshare.31281223). High-magnification imaging of the internal matrix revealed bacterial morphotypes in close physical association with the fungal structures (Fig. 4d through f and h through l; Fig. S6, https://doi.org/10.6084/m9.figshare.31281223). Bacteria were observed adhering directly to the hyphal surface (Fig. 4d through f and h through l; Fig. S6, https://doi.org/10.6084/m9.figshare.31281223) or embedded within the surrounding ECM (Fig. 4e and f; Fig. S6, https://doi.org/10.6084/m9.figshare.31281223), providing visual confirmation of the intimate co-localization implied by our microbiome sequencing.

These SEM findings fundamentally redefine the aspergilloma from a mere fungal aggregate to a structured, polymicrobial biofilm. The physical proximity of bacteria and fungi within a shared ECM scaffold creates a microenvironment conducive to metabolic exchanges and chemical warfare, as revealed by our metabolomic and metatranscriptomic analyses. This intricate architecture likely contributes to the therapeutic recalcitrance and chronicity of the infection by providing a physical barrier against host immune effector cells and antimicrobial agents, while simultaneously facilitating direct and indirect microbe-microbe interactions.

### Metatranscriptomics of the bacterial/fungal populations

To define the metatranscriptional landscape of bacterial co-colonization in *A. fumigatus* fungal balls (with >95% colonization), we focused our analysis on seven representative patients from the original cohort of 34. This final selection was based on the following two criteria: to capture the spectrum of bacterial colonization states and to ensure analytical feasibility by avoiding samples with technical challenges in transcript alignment and annotation. The selected patients and their primary colonizers are A8 and A30 (>95% *H*. *influenzae*); A25, A32, and A61 (>80% *P*. *aeruginosa*); A33 (a mixed community dominated by >40% *P*. *aeruginosa*); and A22 (>95% *N*. *oralis*).

**Fig 5:**
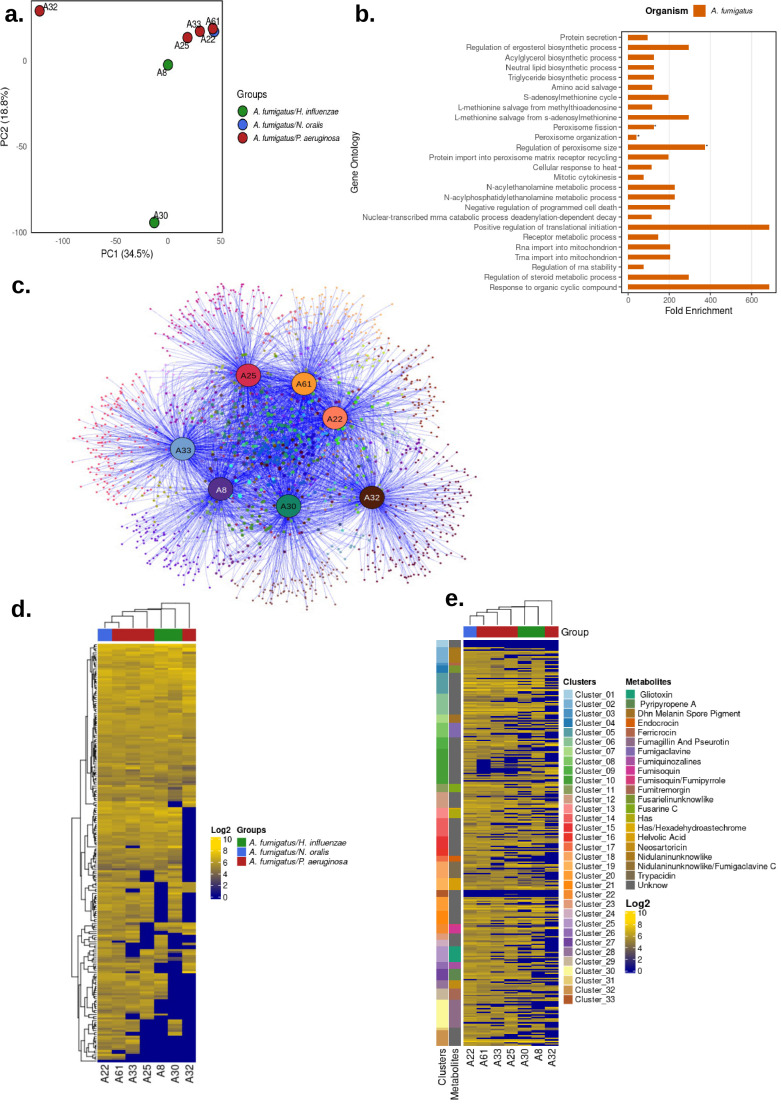
Transcriptomics of *A. fumigatus* indicates elevated expression of genes encoding virulence determinants and secondary metabolites. (**a**) Principal component analysis based on global gene expression obtained by metatranscriptomics of seven aspergilloma samples. Each point represents a sample, colored according to the clustering determined by expression similarity (FPKM). The separation between groups indicates distinct transcriptional patterns among the fungal isolates. (**b**) GO enrichment analysis of differentially expressed genes. The *x*-axis represents fold enrichment, and the *y*-axis represents GO categories. Adjusted significance values are indicated by “*” for FDR < 0.05. (**c**) Venn networking was constructed from the 500 most expressed genes of each sample. The overlap between sets reveals a conserved core of genes highly expressed in multiple isolates, representing the core transcriptional signature of *A. fumigatus* during infection. (**d**) Heatmap of A. fumigatus virulence genes, with expression values converted to log₂ (FPKM). Colors range from blue (low expression) to yellow (high expression), and hierarchical clustering was performed using the Euclidean method. (**e**) Heatmap of *A. fumigatus* secondary metabolite biosynthetic clusters (BGCs). Lines represent 33 distinct clusters, each classified by chemical class (polyacetides, non-ribosomal peptides, alkaloids, terpenes, and siderophores). Color intensity reflects gene expression (FPKM converted to log₂).

PCA analysis shows a high correlation between *A. fumigatus* gene expression profiles in the patients colonized by *P. aeruginosa* (A25, A33, and A61), *H. influenzae* (A8), and *N. oralis* (A22) (Fig. 5a). In patient A32 (colonized by *P. aeruginosa*), there is no correlation, which could be due to the fungal ball having less than 75% *P. aeruginosa* colonization (Fig. 5a). It remains to be determined why there is a lack of correlation in patient A30 (colonized by *H. influenzae*) (Fig. 5a). Gene ontology (GO; https://www.geneontology.org/) categorization of the *A. fumigatus* genes expressed in the fungal balls showed an enrichment for protein secretion, lipid metabolism, S-adenosylmethionine and L-methionine pathways, peroxisome function, cellular response to heat, and positive regulation of translational initiation (Fig. 5b).

An exploration of the overlap among the 500 most highly expressed genes in *A. fumigatus* using a Venn network revealed patterns of proximity between samples, particularly among those coinfected with opportunistic bacteria, suggesting that the presence of different bacterial species induces distinct fungal gene expression profiles (Fig. 5c; see Table S5 at https://doi.org/10.6084/m9.figshare.31281223). We observed that samples associated with *P. aeruginosa* (A25, A32, A33, and A61) formed a tight cluster, indicating convergence in the expression of key genes involved in microenvironmental adaptation. Similarly, samples coinfected with *H. influenzae* (A8 and A30) and *N. oralis* (A22) clustered closely together in the network, suggesting that coinfection with specific bacterial species can elicit distinct fungal responses (Fig. 5c). Other samples, with no detectable presence of opportunistic bacteria or less frequent coinfections, showed a more dispersed distribution, highlighting greater variability in gene expression in the absence of consistent bacterial stimuli (Fig. 5c). This arrangement reinforces the idea that the global expression profile of *A. fumigatus* is not homogeneous across patients but tends to organize into distinct patterns based on the type of interaction with the associated microbiome.

Furthermore, we identified sets of genes exclusively expressed under specific conditions (see Table S5 at https://doi.org/10.6084/m9.figshare.31281223), restricted to certain samples, which may reflect particular responses of the fungus to interactions with specific microorganisms or host conditions. This pattern suggests that bacterial coinfection not only modulates the global expression of *A. fumigatus* but also directs the activation of unique pathways, possibly related to pathogenicity and infection persistence.

We evaluated each fungal ball for the expression of 207 genes previously associated with virulence in *A. fumigatus* (see Table S5 at https://doi.org/10.6084/m9.figshare.31281223) (27–30). In patient fungal balls colonized with *P. aeruginosa*, 51% (A32), 81% (A25), 90% (A33), and 98% of these genes were expressed at different levels (Fig. 5c; Table S5, https://doi.org/10.6084/m9.figshare.31281223). In fungal balls colonized with *H. influenzae*, there are 73% (A8) and 74% (A30) of gene expression, while in *N. oralis,* 99% of the genes are expressed (Fig. 5c; Table S5, https://doi.org/10.6084/m9.figshare.31281223). We also analyzed the expression of 33 curated secondary metabolite gene clusters present in the reference *A. fumigatus* Af293 genome (Table S5, https://doi.org/10.6084/m9.figshare.31281223) (31). As previously shown in the metabolomics analysis (Fig. 5d; Table S5), the fumagillin and fumigaclavine C gene clusters are expressed in most patients (Fig. 5d; Table S5, https://doi.org/10.6084/m9.figshare.31281223). Although gliotoxin, a powerful immunosuppressor and antibacterial inhibitor (32), is not detected in the metabolomics data, the gene cluster is also expressed in most patients (Fig. 5d; Table S5, https://doi.org/10.6084/m9.figshare.31281223). The same is observed for helvolic acid, a potent antifungal and antibacterial compound produced by *A. fumigatus* (Fig. 5d; Table S5, https://doi.org/10.6084/m9.figshare.31281223) (33). These results suggest *that A. fumigatus* expresses most of the genes essential for virulence and that a selected subset of genes is involved in the production of secondary metabolites.

**Fig 6:**
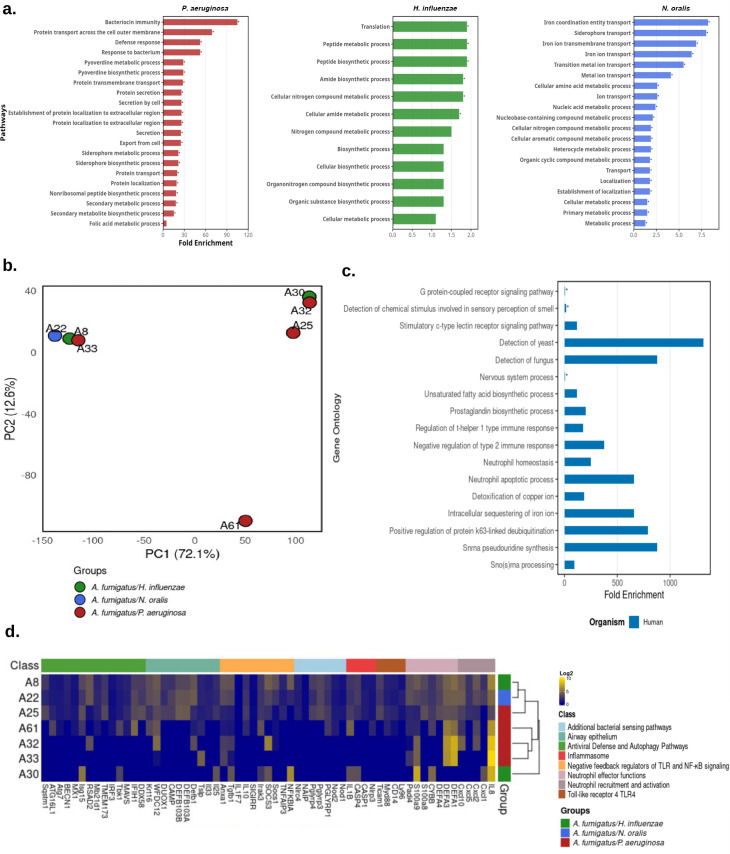
Bacterial functional enrichment and differential gene expression of the human host in pulmonary aspergilloma. (**a**) Enrichment analysis of bacterial metabolic pathways, highlighting *P. aeruginosa* (red), *H. influenzae* (blue), and *N. oralis* (green). Each point represents an enriched pathway, and statistical significance was corrected for multiple testing (FDR < 0.05, indicated by “*”). (**b**) Principal component analysis (PCA) based on global gene expression of the human host for seven samples. The separation of groups suggests differences in the expression profile between patients, potentially related to the immune response and the associated microbial load. (**c**) Gene ontology enrichment of differentially expressed genes in the host. The most significant categories (FDR < 0.05, marked by “*”). (**d**) Heatmap of the main immunological genes expressed in the host, with expression values normalized to log₂ (FPKM). Colors range from blue (lowest expression) to yellow (highest expression). Hierarchical clustering was performed using the Euclidean method, and genes were classified into functional categories related to the inflammatory response.

GO analysis of the bacterial-expressed genes showed enrichment for iron assimilation, secretion, metabolism of organic compounds, production of secondary metabolites, bacteriocin immunity, and the cellular aromatic compound metabolic process (Fig. 6a; see Table S6 at https://doi.org/10.6084/m9.figshare.31281223). The three bacterial species not only have specific GO categories but also share a few GO categories, such as iron metabolism (*P. aeruginosa* and *N. oralis*), cellular nitrogen compound metabolic process (*H. influenzae* and *N. oralis*), organic compound metabolic process (*N. oralis* and *H. influenzae*), and protein secretion and transport (*P. aeruginosa* and *N. oralis*) (Fig. 6b; Table S6, https://doi.org/10.6084/m9.figshare.31281223). In conclusion, the functional categorization of the bacterial metatranscriptome reveals a dynamic, antagonistic biofilm environment. The microbial consortium is not merely co-existing but is engaged in a multifaceted interplay characterized by active nutrient acquisition, robust biosynthesis, and a sophisticated chemical warfare centered on iron scavenging and the production of antimicrobial secondary metabolites.

### Host genes from metatranscriptomic profiling reveal a dysregulated and multifaceted innate immune response in *A. fumigatus* fungal balls co-colonized by bacteria

PCA analysis shows no correlation between the patient fungal balls colonized by different bacterial species and human gene expression (Fig. 6c; see Table S7 at https://doi.org/10.6084/m9.figshare.31281223). This suggests that the human response is not specific to a single pathogen but is most likely dependent on the interaction between the fungus and the bacterial species, or on a particular genetic/phenotypic patient background. GO categorization of the human genes expressed in the fungal balls showed an enrichment for G protein-coupled receptor signaling pathway, detection of chemical stimulus involved in sensory perception of smell and fungi, unsaturated fatty acid biosynthetic process, prostaglandin biosynthetic process, negative regulation of type 2 immune response, neutrophil homeostasis, and intracellular sequestering of iron ion (Fig. 6d).

We observed 1,226 human genes involved in the immunological response as expressed in the fungal balls (https://www.innatedb.com/; tFig. 6c; see Table S7 a https://doi.org/10.6084/m9.figshare.31281223). A close look at the expression of 61 of these genes defined several immunological responses to the fungal balls (Fig. 6d; Table S7, https://doi.org/10.6084/m9.figshare.31281223). We observed significant upregulation of genes encoding potent neutrophil chemoattractants, including *IL8* (CXCL8), *CXCL1*, *CXCL2*, *CXCL5*, and *CXCL10* (Fig. 6d; Table S7, https://doi.org/10.6084/m9.figshare.31281223). Furthermore, transcripts indicative of neutrophil effector functions were highly abundant, such as the alpha-defensins *DEFA1*, *DEFA3*, and *DEFA4*; the NADPH oxidase component *CYBB* (NOX2); the heterodimer S100A8/S100A9 (calprotectin), essential for phagosomal oxidative burst; and *PADI4*, a key enzyme in the formation of neutrophil extracellular traps (NETs) (Fig. 6d; Table S7, https://doi.org/10.6084/m9.figshare.31281223). This pattern suggests sustained and robust neutrophil recruitment, whose antimicrobial arsenal may contribute to collateral tissue damage within the confined space of the fungal ball.

The presence of gram-negative bacteria was reflected in a pronounced transcriptional signature of Toll-like receptor 4 (TLR4) pathway activation. Key genes involved in TLR4 signaling, including *TLR4* itself, its co-receptors *LY96* (MD-2) and *CD14*, and the adaptor molecules *MYD88* and *TICAM1* (TRIF), were highly expressed (Fig. 6d; see Table S7 at https://doi.org/10.6084/m9.figshare.31281223). Concurrently, we detected strong evidence of inflammasome activation, with elevated expression of NLRP3, CASP1, CASP4, and their key product *IL1B* (Fig. 6d; Table S7, https://doi.org/10.6084/m9.figshare.31281223). Additional bacterial sensing pathways were also engaged, as indicated by the expression of NOD1/NOD2; peptidoglycan recognition proteins *GLYRP1*, *PGLYRP3*, and *PGLYRP4*; and the NAIP/NLRC4 inflammasome complex (Fig. 6d; Table S7, https://doi.org/10.6084/m9.figshare.31281223). This indicates that bacterial components are a significant stimulus for the hyper-inflammatory state.

A striking feature of the data was the simultaneous high expression of both pro-inflammatory and anti-inflammatory regulators. Alongside inflammatory cytokines, we observed a significant upregulation of multiple negative feedback regulators of TLR and NF-κB signaling, including *NFKBIA* (IκBα), *TNFAIP3* (A20), *SOCS1*, *SOCS3*, *IRAK3*, and the decoy receptor *SIGIRR* (Fig. 6d; Table S7, https://doi.org/10.6084/m9.figshare.31281223). Anti-inflammatory cytokines such as *IL-10, IL-37*, and *TGFB1* were also transcribed, alongside the pro-resolving mediator *ANXA1* (Annexin A1) (Fig. 6d; Table S7, https://doi.org/10.6084/m9.figshare.31281223). This co-expression suggests that the host is actively attempting to dampen the inflammatory response but failing to achieve resolution despite persistent biofilm stimulation.

The transcriptome indicated that the airway epithelium is not a passive barrier but an active participant in the immune response. We found a high expression of epithelial-derived alarmins (*IL25*, *IL33*, and *TSLP*) that activate innate lymphoid cells and a suite of antimicrobial peptides (*DEFB1*, DEFB103A/B, and CAMP/LL-37) (Fig. 6d; see Table S7 at https://doi.org/10.6084/m9.figshare.31281223). The epithelium also appeared to mount a direct oxidant defense via DUOX1 and was a significant source of the neutrophil chemoattractant IL-8. The expression of stress-response genes like *WFDC12* and *KRT16* further underscores a state of epithelial activation and remodeling (Fig. 6d; Table S7, https://doi.org/10.6084/m9.figshare.31281223).

Unexpectedly, we observed a strong transcriptional signature of pathways typically associated with antiviral immunity. This included the RIG-I-like receptor (RLR) pathway (DDX58/RIG-I, IFIH1/MDA5, *MAVS*, *TBK1*, and *IRF3*) and the cGAS-STING pathway (TMEM173/STING and MB21D1/cGAS), leading to the expression of interferon-stimulated genes (RSAD2/Viperin, *ISG15*, and *MX1*) (Fig. 6d; see Table S7 at https://doi.org/10.6084/m9.figshare.31281223). Additionally, core components of the autophagy machinery (*BECN1*, *ATG7*, *ATG16L1*, and SQSTM1/p62) were upregulated, suggesting their involvement in pathogen clearance (xenophagy) and immune regulation (Fig. 6d; Table S7, https://doi.org/10.6084/m9.figshare.31281223). This indicates a broader role for these pathways in polymicrobial biofilm defense, potentially through the sensing of microbial nucleic acids.

## DISCUSSION

This study provides an unprecedented, multi-omics portrait of the biofilm pulmonary fungal ball. It moves beyond the classical view of a monospecies mycetoma to reveal it as a complex, integrated, and dysregulated polymicrobial ecosystem. We report the first in-depth, multi-omics investigation of the biofilm environment in CPA and lung fungus balls, delineating the constituent microbial communities and their concomitant production of metabolites and transcripts alongside those of the human host. By integrating microbiome, metabolome, and metatranscriptome data from a uniquely large cohort of clinical specimens, we demonstrate that the fungal ball is a site of intense cross-kingdom interaction, where bacteria and fungi engage in a dynamic interplay of competition and co-existence, collectively driving a host immune response that is both hyperactive and futile.

Our initial characterization of the cohort confirms the heterogeneous clinical presentation of fungal ball disease but reveals a striking microbial convergence. Despite diverse patient histories, *A. fumigatus* dominates the fungal niche, while a limited set of opportunistic bacteria, notably *P. aeruginosa* and *H. influenzae*, are recurrent partners. *A. fumigatus* is the most common filamentous fungus detected in chronic respiratory diseases and is almost always accompanied by bacteria, especially gram-negative bacilli, such as *P. aeruginosa* and others (15, 20, 34, 35). In a previous study, clinical isolates were obtained from an Indonesian collection and from the sputum of post-tuberculosis patients with suspected CPA (36). The isolates were identified using a combination of DNA analyses. Among the patients, 29 (49%) met the diagnostic criteria for CPA, and 54% were *A. fumigatus*-positive (36). In our study, the positive correlation between bacterial diversity and fungal ball diameter suggests that a more complex community can be supported in larger lesions or that a more diverse bacterial influx promotes lesion expansion. The specific association of *P. aeruginosa* with *A. fumigatus* points to a remarkably stable and influential interkingdom relationship within this niche, which is commonly observed in the lungs of cystic fibrosis patients (15, 20, 34, 35).

**Fig 7:**
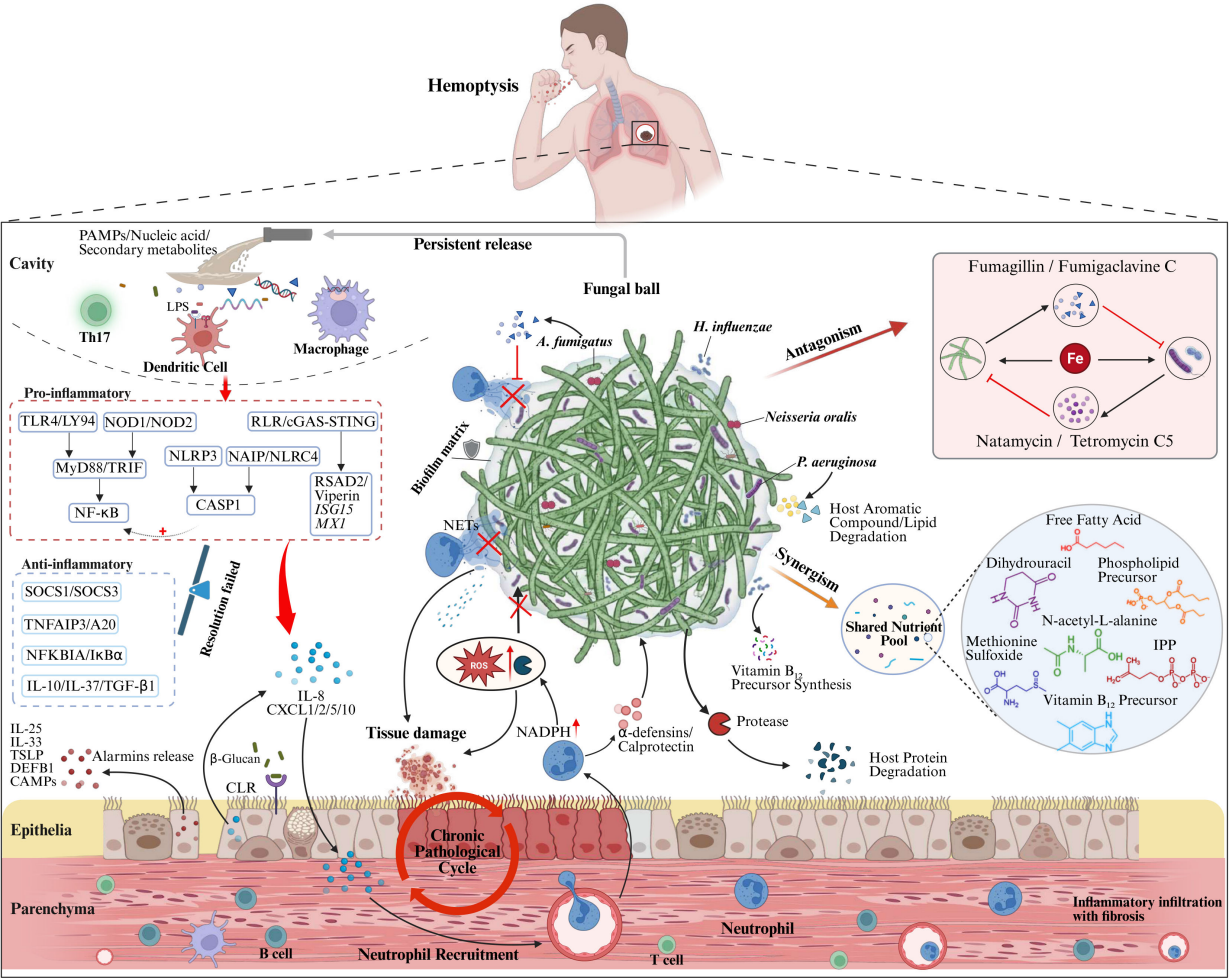
Integrated model of the host-microbe interplay and immune-metabolic adaptations in chronic pulmonary aspergillosis (CPA) fungal balls. The schematic illustrates the key pathways and components identified through multi-omics profiling. The core of the fungal ball is characterized by *A. fumigatus* hyphae and a diverse bacterial microbiome (e.g., *H. influenzae*, *P. aeruginosa*, and *N. oralis*), which contributes to a complex inflammatory milieu. Host immune recognition via PAMP sensing (e.g., nucleic acids) triggers pro-inflammatory signaling cascades (e.g., NF-κB and NLRP3), leading to the recruitment and activation of neutrophils, macrophages, dendritic cells, and T cells. This is accompanied by a robust cytokine/chemokine response (e.g., IL-1β, IL-8, IL-17). Concurrent metabolic adaptations are observed, including alterations in lipid metabolism (e.g., triglycerides and phospholipids) and in nutrient availability (e.g., iron, zinc, and vitamin B). The model also highlights fungal strategies for persistence, such as protease release, siderophore-mediated iron acquisition, and the induction of host anti-inflammatory feedback mechanisms (e.g., IL-10, IL-37, and TGF-β1) that may contribute to chronicity. The overall process can lead to tissue damage and clinical manifestations such as hemoptysis. Biorender was used to draw the figure.

The metabolomic data offer a direct chemical snapshot of this ecosystem, revealing a “cross-kingdom metabolic network” (Fig. 7). The environment is characterized by intense nutrient mining, as evidenced by fungal proteolysis and bacterial degradation of aromatic host compounds. This creates a shared pool of metabolites that fuels the entire community, illustrating a saprophytic tripartite system in which host tissue serves as the ultimate carbon source. Simultaneously, the metabolome is saturated with the biosynthetic products of biological competition. We detected *A. fumigatus* secondary metabolites, such as fumagillin and fumiclavine C30, and bacterial antibiotics, such as polyenes, alongside molecular signatures of competition for iron (Fig. 7). Fumagillin covalently binds Methionine Aminopeptidase 2 (MetAP2), causing irreversible inhibition of the enzyme, disrupting protein processing, leading to cell cycle arrest (G1 phase) and inhibition of proliferation; it is a potent anti-angiogenesis agent, affecting the migration and the proliferation ability of the A549 pulmonary cells, and the viability and proliferation ability of the RAW 264.7 macrophages (37). The mechanism of action of fumigaclavine C is not fully elucidated. However, it is capable of local immunosuppression (inhibiting pro-inflammatory cytokine production), systemic manipulation via dopamine receptor agonism, and potential disruption of bacterial quorum sensing (33, 38). This constant antagonism likely shapes community structure, selecting for robust, well-defended members.

Essentially, the metatranscriptomic data confirm that this metabolic activity is driven by active gene expression from all residents. *A. fumigatus* exhibits a transcriptional profile of stress adaptation, active metabolism, and high virulence gene expression, which is perceptibly modulated by the co-colonizing bacterial species (39–41). The clustering of fungal gene expression by bacterial partners demonstrates that interkingdom signaling or competition is a key determinant of fungal physiology *in vivo*. The consistent expression of virulence and secondary metabolite gene clusters, even for compounds like gliotoxin, which are not detected metabolically, shows the fungus is perpetually poised for pathogenicity and defense. On the bacterial side, the transcriptional enrichment for iron acquisition, secretion systems, and secondary metabolite production directly mirrors the metabolomic findings, confirming an active, antagonistic, and resource-competitive bacterial community.

The host’s response to this polymicrobial consortium is a masterclass in dysregulated immunity (42, 43) (Fig. 7). Our human metabolomics data support a paradigm in which the fungal ball is not a passive obstruction but a dynamic, pathological entity that actively drives a state of chronic and damaging inflammation. We propose that the biofilm functions as a persistent inflammatory engine, continuously exposing the host immune system to a complex array of fungal and bacterial antigens (Fig. 7). This is evidenced by the sustained elevation of inflammatory eicosanoids, including leukotriene C4 and specific prostaglandins (PGE3 and PGD2-1-glyceryl ester), indicative of persistent neutrophil and macrophage activation. The tissue damage not only perpetuates the inflammatory cascade but also liberates host-derived nutrients that may subsequently fuel the microbial community, thereby reinforcing the biofilm’s stability and resilience. This feed-forward loop underscores the biofilm’s role as the central orchestrator of its own persistence and the associated host pathology. Interestingly, two reports on the metabolomics of fungal ball sinusitis and paranasal sinuses in several patients (24, 25) also reported increased production of lipid classes such as lysophosphatidylcholine (LPC) O-18:0, LPC O-20:1, and LPE O-20:1, as well as glycerophospholipids, and sphingolipids, including sphingosine 1-phosphate (S1P) and related products, diacylglycerol, and sphingomyelin (SM), suggesting that its metabolites are associated with mucosal and bony inflammation (24, 25).

The human transcriptome corroborates the metabolomics data, revealing a state of chronic, neutrophilic inflammation, driven by the simultaneous detection of fungal and bacterial components via multiple sensory pathways, including TLRs, NLRs, and, unexpectedly, RLRs and cGAS-STING (Fig. 7). This suggests that microbial nucleic acids released within the biofilm contribute to the hyper-inflammatory state. Dysregulation of this pathway has been linked to several chronic lung diseases, such as cystic fibrosis, COPD, idiopathic pulmonary fibrosis, and asthma (44). The concurrent, high-level expression of both potent pro-inflammatory mediators and a comprehensive suite of anti-inflammatory and resolution signals is particularly telling. It depicts a host caught in a futile cycle, simultaneously launching a destructive, inflammatory assault and desperately trying to restrain it, unable to resolve the response in the face of the persistent biofilm stimulus.

The profound upregulation of a suite of neutrophil chemoattractants (e.g., IL8, CXCL1, and CXCL2) and the high abundance of transcripts encoding neutrophil antimicrobial effectors (e.g., defensins, calprotectin, and PADI4) paint a picture of continuous neutrophil recruitment and activation. While this response is intended to clear the infection, in the confined, avascular space of a fungal ball, it is likely a primary driver of the tissue damage and bronchiectasis characteristic of this disease (Fig. 7). The neutrophils appear to be “trapped” in a cycle of activation, releasing their cytotoxic contents but failing to eradicate the embedded biofilm. The host’s sensory apparatus is on high alert, with clear signatures of engagement against both fungal and bacterial components. The pronounced TLR4 pathway activation, alongside NOD1/NOD2 and inflammasome signaling (NLRP3 and NLRC4), reflects a robust response to gram-negative bacteria like *P. aeruginosa* and *H. influenzae* (45, 46) (Fig. 7). This bacterial footprint is a critical finding, as it positions the fungal ball not as a purely mycotic process but as a polymicrobial entity that provokes a broader, more intense inflammatory reaction than fungus alone might elicit. The concurrent high expression of cytokines like IL-1β and IL-10, and of activators like MYD88 alongside negative regulators like TNFAIP3 (A20) and SOCS proteins, indicates a host desperately trying to restrain its own destructive inflammatory response. This unresolved state is a hallmark of chronic inflammatory diseases. These data suggest that constant stimulation by the polymicrobial biofilm creates an insurmountable barrier to resolution, leading to a pathologic equilibrium in which inflammation persists without clearance.

The airway epithelium emerges as a key orchestrator in this response (Fig. 7) (47). Its active role is evidenced by the production of alarmins (IL-25, IL-33, and TSLP) (48), antimicrobial peptides (49), and oxidant defenses (DUOX1). This positions the epithelium not merely as a barrier but as a central signaling hub that amplifies and sustains the immune response, potentially contributing to the type 2 immune skewing (50, 51) observed through the negative regulation of type 2 immunity GO term (Fig. 7). In conclusion, our metatranscriptomic profiling reveals the *A. fumigatus* fungal ball as a site of profound immune dysregulation. The host mounts a multifaceted but futile assault, characterized by a neutrophilic onslaught, hyperactivation of diverse pathogen-sensing pathways, and a failed resolution program, all set against the backdrop of an activated and stressed epithelium. The epithelium contributes actively to both antimicrobial defense and inflammatory signaling. However, the persistent biofilm structure likely renders this cellular response ineffective, leading to a pathological stalemate. The presence of bacteria amplifies this response, shifting the paradigm of the fungal ball from a passive mycetoma to an active polymicrobial driver of chronic inflammation.

Synthesizing these findings, we propose a refined model of the fungal ball as a self-sustaining, pathological entity. The core is a polymicrobial biofilm where *A. fumigatus* provides the structural scaffold (Fig. 7). The associated bacteria, through their metabolic activities and ongoing antagonism, create a nutrient-rich, chemically aggressive environment that promotes fungal virulence and biofilm stability. This thriving microbial community continuously sheds antigens and microbe-associated molecular patterns, stimulating a massive and destructive neutrophilic influx from the host (Fig. 7). The host response is sufficient to cause significant collateral tissue damage—explaining symptoms like hemoptysis and bronchiectasis—but insufficient to clear the infection. The fungal ball thus persists as an inflammatory driver of lung destruction. A limitation of this study is the inherent challenge of assigning absolute causation from human samples. While our multi-omics approach provides an unprecedented systems-level view of the fungal ball ecosystem, certain limitations offer avenues for future investigation. First, as an observational clinical study, the compelling associations we describe—such as the metabolic division of labor between microbes and the host’s dysregulated immune signature—warrant functional validation of their causal roles in pathogenesis. Future work employing *in vitro* polymicrobial biofilm models and animal models of co-infection will be essential to directly test these hypotheses and establish mechanistic causality. Second, our bulk analyses, although comprehensive, integrate signals across the entire fungal ball. The spatial organization of these cross-kingdom interactions remains a critical open question. Techniques such as spatial transcriptomics and imaging mass spectrometry (e.g., MALDI-TOF) on tissue sections could map the physical proximity of microbial members to their metabolic products, revealing the micro-architecture of this polymicrobial city state.

In conclusion, our work deconstructs the fungal ball ecosystem, revealing it as a nexus of interkingdom conflict and collaboration that drives disease persistence. These findings suggest that therapeutic strategies aimed solely at pathogen eradication may be insufficient; adjunctive therapies that target the host response, particularly by breaking the cycle of neutrophilic inflammation and promoting resolution, could be essential for mitigating the long-term tissue damage associated with this debilitating infection. Future efforts must consider the ecosystem as a whole, exploring interventions that disrupt the polymicrobial synergy—for instance, by interfering with cross-kingdom communication, nutrient sharing, or the shared battle for iron—to break the cycle of inflammation and tissue damage that defines this debilitating disease.

## MATERIALS AND METHODS

### Study population

This prospective study enrolled 62 Chinese patients diagnosed with chronic pulmonary aspergillosis (CPA) at Shanghai Pulmonary Hospital between January 2020 and December 2024. One patient (A49) was excluded post-operatively upon pathological diagnosis of active pulmonary tuberculosis, which was an exclusion criterion. Consequently, 61 patients were included in the final multi-omics analysis. Key clinical and demographic data, including age, sex, underlying lung disease, smoking history, and prior antifungal treatment history, were prospectively collected.

Patient eligibility was rigorously assessed based on the 2016 diagnostic guidelines for chronic pulmonary aspergillosis (simple aspergilloma or chronic cavitary pulmonary aspergillosis), jointly issued by the European Society for Clinical Microbiology and Infectious Diseases (ESCMID), the European Respiratory Society (ERS), and the European Confederation of Medical Mycology (ECMM). To be included, patients were required to meet all of the following criteria. (i) Clinical evidence included presence of respiratory and/or constitutional symptoms (e.g., cough, hemoptysis, dyspnea, weight loss, or fatigue) for at least 3 months. (ii) Radiological evidence included chest computed tomography (CT) confirming an intracavitary fungal ball (aspergilloma) within one or more pulmonary cavities. For patients with CCPA, additional evidence of radiological progression over at least 3 months was necessary (e.g., expansion of existing cavities, new cavity formation, or increasing peri-cavitary infiltrates). (iii) Microbiological or immunological evidence included at least one positive finding, including elevated serum *Aspergillus*-specific IgG antibodies, a positive serum or bronchoalveolar lavage (BAL) galactomannan (GM) antigen test, or direct detection of *Aspergillus* spp. from a respiratory sample (sputum, BAL fluid, or biopsy) by microscopy or culture. (iv) Clinical decision for surgery meant being scheduled to undergo surgical resection (video-assisted thoracoscopic surgery [VATS] or thoracotomy) for disease management, primarily for indications such as significant or recurrent hemoptysis.

Patients were excluded if any of the following conditions were present: a diagnosis of invasive aspergillosis (IA); severe immunocompromise, defined as neutropenia (<500 cells/µL), receipt of a hematopoietic stem cell or solid organ transplant within the last year, or active treatment with high-dose corticosteroids (>20 mg/day prednisone equivalent for >2 weeks) or other potent immunosuppressive agents; active pulmonary tuberculosis or nontuberculous mycobacterial infection confirmed by microbiology; confirmed lung malignancy within the resected lesion; or inability to provide informed consent.

### Sample collection and processing

For all included patients, fungal ball samples were obtained intraoperatively from the surgically resected lung lobes. The procedure was conducted by thoracic surgeons, and samples were handled under sterile conditions to minimize contamination. Within 30 min of resection, the fungal ball was macroscopically identified and meticulously excised from the surrounding lung tissue. Each specimen was immediately divided into multiple aliquots, placed into sterile, nuclease-free cryotubes, flash-frozen in liquid nitrogen, and stored at −80°C until multi-omics analysis.

### Histopathological staining

Surgical specimens of the aspergilloma and surrounding lung tissue, obtained via VATS or thoracotomy lobectomy, were fixed in 10% neutral buffered formalin for 24–48 h and subsequently embedded in paraffin; 3-µm-thick sections were cut from the formalin-fixed, paraffin-embedded (FFPE) blocks and mounted on adhesive slides. For staining, the sections were deparaffinized using an eco-friendly de-waxing solution (Servicebio, G1128) and rehydrated through a graded ethanol series to distilled water. HE staining was performed using a high-definition staining kit (Servicebio, G1076) following the manufacturer’s protocol. Briefly, rehydrated sections were pre-treated for 1 min, stained with hematoxylin for 3–5 min, washed, differentiated in acid alcohol, and blued. Sections were then counterstained with eosin for 15 s, dehydrated through graded ethanol and xylene, and mounted using neutral balsam. PAS staining was conducted using a commercial kit (Servicebio, G1008). Rehydrated sections were oxidized in 0.5% periodic acid (solution A) for 25–30 min at room temperature, protected from light. After a 5-min wash in running water, slides were immersed in Schiff’s reagent (solution B) for 10–15 min. Sections were then washed and counterstained with hematoxylin (solution C) for 30 s, followed by differentiation and bluing. Slides were dehydrated and mounted as described. GMS staining was performed using a kit (Servicebio, G1059). Rehydrated sections were oxidized in 5% chromic acid (Solution B) for 15–20 min and washed thoroughly with distilled water. A working methenamine silver solution was prepared from solutions C, D, and E (G1059 kit) and pre-warmed to 56°C–59°C. Slides were incubated in this solution, and staining progression was monitored microscopically starting at 40 min and checking every 10 min thereafter until fungal elements turned black. After washing in distilled water, sections were briefly treated with 0.2% gold chloride (component of solution F, as per G1059 protocol) to tone the reaction, washed, treated with 2% sodium thiosulfate, and finally counterstained with Light Green (solution F) for 20 s. Sections were then dehydrated and mounted.

All stained sections were examined and imaged using a Nikon Eclipse E100 upright microscope equipped with a Nikon DS-U3 imaging system, and digital slides were reviewed using CaseViewer software (3DHISTECH).

### Total genomic DNA extraction

Total genomic DNA was extracted from ~50 mg of fungal ball samples using a modified cetyl trimethylammonium bromide (CTAB) method. Samples were homogenized in CTAB extraction buffer and incubated with lysozyme (10 mg/mL) in a water bath at 65°C for 2 h. The lysate was purified using phenol:chloroform:isoamyl alcohol (25:24:1) and chloroform:isoamyl alcohol (24:1). DNA was precipitated with isopropanol, washed twice with 75% ethanol, and re-dissolved in deionized water. An RNase A digestion step was performed at 37°C for 15 min. DNA concentration and purity (A260/A280 ratio) were assessed using a NanoDrop One spectrophotometer (Thermo Fisher Scientific), and integrity was verified using 1% agarose gel electrophoresis. DNA was diluted to 1 ng/μL for sequencing.

### Total RNA extraction

Total RNA was extracted from ~50 mg of fungal ball samples using TRIzol Reagent (Invitrogen). Samples were homogenized in 1 mL of TRIzol with 3–4 sterile steel beads (6.0 m/s, 40 s, FastPrep-24) using a FastPrep-24 homogenizer (MP Biomedicals) at 6.0 m/s for 40 s. After homogenization, RNA extraction was performed according to the manufacturer’s protocol. To enhance RNA precipitation, 2 μL of GlycoBlue Coprecipitant (Invitrogen) was added. The extracted RNA was dissolved in RNase-free water. RNA concentration and purity were measured using a NanoDrop One spectrophotometer. RNA integrity was assessed using an Agilent 2100 Bioanalyzer, and only high-quality RNA samples with an RNA integrity number (RIN) >7.0 were used for library preparation.

### Amplicon sequencing (16S rRNA gene and ITS)

The V3-V4 hypervariable region of the 16S rRNA gene and the fungal ITS1 region were amplified. For all samples, the 16S primer pair was 341F (5′-CCTAYGGGRBGCASCAG-3′) and 806R (5′-GGACTACNNGGGTATCTAAT-3′), and the ITS primer pair was ITS1F (5′-CTTGGTCATTTAGAGGAAGTAA-3′) and ITS2 (5′-GCTGCGTTCTTCATCGATGC-3′). PCRs were performed in 15 μL volumes containing Phusion High-Fidelity PCR Master Mix (New England Biolabs), 2 μM of each primer, and 10 ng of template DNA. Thermal cycling conditions were as follows: 98°C for 1 min; 30 cycles of 98°C for 10 s, 50°C for 30 s, and 72°C for 30 s; and a final extension at 72°C for 5 min. PCR products were purified using gel electrophoresis, and sequencing libraries were prepared using the TruSeq DNA PCR-Free Sample Preparation Kit (Illumina). Libraries were sequenced on an Illumina NovaSeq 6000 platform to generate 250 bp paired-end reads.

### Metatranscriptomic sequencing

Metatranscriptomic libraries were constructed from 10–100 ng of total RNA using the AccuNext Stranded Single Cell & Low Input RNA-seq Library Prep Kit for Illumina (AccuraBio, China). The protocol included the depletion of both prokaryotic (16S/23S) and eukaryotic (18S/28S) ribosomal RNA. The enriched RNA was fragmented and reverse-transcribed using random primers. Second-strand cDNA synthesis incorporated dUTP to ensure strand specificity. The resulting double-stranded cDNA was ligated with adapters containing unique dual indexes (UDIs) and amplified with an optimized PCR cycle count (12–15 cycles).

Library concentration was quantified using a Qubit 4.0 Fluorometer, and size distribution was verified using an Agilent Bioanalyzer 2100. Equimolar pools of the final libraries were sequenced on an Illumina NovaSeq 6000 platform, generating 150 bp paired-end reads.

### Metabolomic analysis (LC-MS/MS)

Metabolites were extracted from ~20 mg of fungal ball samples using an 80% methanol solution pre-cooled to −20°C. Samples were homogenized with ceramic beads, vortexed, and incubated at −20°C for 1 h. After centrifugation (14,000 × *g*, 15 min, 4°C), the supernatant was collected and dried under a vacuum. The dried extract was reconstituted in a 50% methanol solution for LC-MS/MS analysis.

Metabolomic profiling was performed using a Thermo Vanquish UHPLC system coupled to a Thermo Q Exactive HF-X mass spectrometer. Separation was achieved on an ACQUITY UPLC HSS T3 column (100 mm × 2.1 mm, 1.8 μm; Waters). The mobile phases consisted of 0.1% formic acid in water (A) and 0.1% formic acid in acetonitrile (B). The mass spectrometer was operated in both positive and negative ion modes.

Raw data files were converted to mzML format and processed using XCMS (v3.12.0) for peak detection, retention time correction, and alignment (52). Metabolite annotation was performed by matching MS/MS spectra against the Human Metabolome Database (HMDB) and METLIN. Approximately 300 metabolites were manually selected based on known metabolites produced by humans, bacteria, and fungi, with synthetic compounds excluded. Principal component analysis was performed in R (v4.3.2) to evaluate clustering and sample distribution. Metabolic pathway enrichment was conducted using MetaboAnalyst 5.0 (53), and pathways with a false discovery rate (FDR) < 0.05 were considered significant.

### Amplicon sequencing and analysis

Raw sequencing reads underwent quality control using fastp (v0.22.0) (54) for adapter trimming, filtering low-quality reads (Phred < 30), and removing sequences with more than 10% ambiguous bases.

Filtered reads were processed in QIIME2 (v2023.5) (55) for demultiplexing, denoising, chimera removal, and generation of operational taxonomic units (OTUs) at 97% similarity. DADA2 (56) was employed for error correction and amplicon sequence variant (ASV) inference, ensuring high-resolution taxonomic discrimination.

Taxonomic classification was performed using the SILVA database (v138.1) for bacterial 16S rRNA and the UNITE database (v8.3) for fungal ITS regions (57, 58). Multiple sequence alignments were generated with MAFFT (v7.515) (59), and phylogenetic trees were reconstructed using IQ-TREE (v2.1.4) (60) with the best-fit substitution model determined automatically by ModelFinder. Interactive visualization and tree annotation were performed with iTOL (v6) (61).

### Metatranscriptomic analysis

Quality control and preprocessing of raw reads were performed as described above. Ribosomal RNA sequences were removed using SortMeRNA (v4.3.6) (62), and potential host-derived contaminants were eliminated by mapping reads against the *Homo sapiens* GRCh38.p14 reference genome (GCF_000001405.40) using Bowtie2 (v2.5.1) (63) with the parameter --very-sensitive.

For eukaryotic transcriptome profiling, high-quality reads were aligned to the *A. fumigatus* Af293 reference genome (GCF_000002655.1) and to the *Homo sapiens* GRCh38.p14 genome using HISAT2 (v2.2.1) (64). Gene expression quantification was carried out with StringTie2 (v2.2) (65) using the official GTF annotation files. Transcript abundances were calculated as fragments per kilobase of transcript per million mapped reads (FPKM).

For bacterial transcriptomic profiling, reads were mapped to the reference genomes of *P. aeruginosa* (GCF_000006765.1), *H. influenzae* (GCF_000027305.1), and *N. oralis* (GCF_045062685.1) using Bowtie2. Gene-level read counts were obtained using featureCounts (v2.0.6) (66), followed by FPKM normalization to allow cross-sample comparison. Functional annotation and pathway enrichment analyses were conducted using iDEP (67). Only the Biological Process (BP) category of the GO database was retained, and pathways showing FDR < 0.05 were considered significantly enriched.

All statistical analyses and visualizations, including dot plots, bar plots, chord diagrams, and PCA, were performed in R (v4.5.1, https://cran.r-project.org/bin/windows/base/)

## Data Availability

The ITSs, 16S rDNAs, and metatranscriptomics were submitted to NCBI under the BioProject ID PRJNA1344265, and the metabolomics data are available at https://zenodo.org/records/17402277.
